# An Internal Defect Detection Algorithm for Concrete Blocks Based on Local Mean Decomposition-Singular Value Decomposition and Weighted Spatial-Spectral Entropy

**DOI:** 10.3390/e25071034

**Published:** 2023-07-09

**Authors:** Xu Tian, Jun Ao, Zizhu Ma, Chunbo Ma, Junjie Shi

**Affiliations:** 1 Research Institute of Optical Communication, School of Information and Communication, Guilin University of Electronic Technology, Guilin 541004, China; 20021101011@mails.guet.edu.cn (X.T.);; 2 Pengcheng Laboratory, Shenzhen 518000, China

**Keywords:** defect detection, signal denoising, laser vibrometry, weighted spatial-spectral entropy

## Abstract

Within the scope of concrete internal defect detection via laser Doppler vibrometry (LDV), the acquired signals frequently suffer from low signal-to-noise ratios (SNR) due to the heterogeneity of the concrete’s material properties and its rough surface structure. Consequently, these factors make the defect signal characteristics challenging to discern precisely. In response to this challenge, we propose an internal defect detection algorithm that incorporates local mean decomposition-singular value decomposition (LMD-SVD) and weighted spatial-spectral entropy (WSSE). Initially, the LDV vibration signal undergoes denoising via LMD and the SVD algorithms to reduce noise interference. Subsequently, the distribution of each frequency in the scan plane is analyzed utilizing the WSSE algorithm. Since the vibrational energy of the frequencies caused by the defect resonance is concentrated in the defect region, its energy distribution in the scan plane is non-uniform, resulting in a significant difference between the defect resonance frequencies’ SSE values and the other frequencies’ SSE values. This feature is used to estimate the resonant frequencies of internal defects. Ultimately, the defects are characterized based on the modal vibration patterns of the defect resonant frequencies. Tests were performed on two concrete blocks with simulated cavity defects, using an ultrasonic transducer as the excitation device to generate ultrasonic vibrations directly from the back of the blocks and applying an LDV as the acquisition device to collect vibration signals from their front sides. The results demonstrate the algorithm’s capacity to effectively pinpoint the information on the location and shape of shallow defects within the concrete, underscoring its practical significance for concrete internal defect detection in practical engineering scenarios.

## 1. Introduction

As an essential component in the construction of load-bearing facilities such as dams, bridges, and buildings, concrete exhibits commendable durability, water resistance, and plasticity. However, during the construction of concrete facilities, cavities or cracks are sometimes formed inside the concrete due to inadequate compaction or seepage of slurry. Besides, environmental erosion and stress fatigue over the long term can further induce defects on the surface and within the structure’s interior. Given the imperceptibility of internal defects to the naked eye, developing effective damage detection techniques becomes imperative to enhance the precision of assessing the concrete’s internal damage condition.

At present, prevailing non-destructive testing (NDT) methodologies for concrete, such as the impact echo method [1,2,3], ground-penetrating radar method [4,5,6], X-ray computed tomography method [7,8], infrared thermography [9,10], and ultrasonic detection method [11,12,13,14], have demonstrated significant efficacy. However, each of these NDT methods bears inherent limitations. For instance, the impact echo method’s efficiency dwindles when conventional inspection instruments are used, such as hammers and iron balls. Although the impact echo scanner (IES) augments the detection efficiency, it necessitates a flat surface on the components under inspection, thereby hampering its applicability to irregular parts [15]. The ground-penetrating radar method is prone to interference arising from physical parameters such as moisture and salt content in concrete and the surrounding environment. Dense steel reinforcements embedded in the concrete can also obstruct the radar signal, complicating the detection of internal defects [16]. X-ray computed tomography has the safety risk of radiation exposure. In addition, due to its high cost and the huge size of the equipment, it is rarely used for infrastructure inspection [17]. Infrared thermography has high requirements of its detection environment, and is easily affected by the detection environment and weather, sometimes resulting in false detection and missing detection [18]. The ultrasonic inspection method mandates the coupling of the sensor to the measured surface, thus requiring scaffolding, sensor anchoring, and wire connections during measurement [19]. This requirement amplifies the inspection’s time and cost constraints, and is sensitive to the installation space of the sensors. 

Laser vibration detection technology is a non-contact, non-destructive testing technology. It has the advantages of high detection bandwidth and spatial resolution and high sensitivity to weak vibrations. With these merits, it has proven successful in detecting surface and shallow defects in metals [20,21], agricultural product quality detection [22], and modal analysis of aerospace equipment [23]. More recently, efforts have been made to employ this technology in the detection of concrete defects [24,25,26].

The typical defect detection algorithms in concrete defect detection are the machine learning algorithm [27,28,29], the synthetic aperture focusing algorithm [30,31], and the modal analysis algorithm [32,33,34], among others. Machine learning-based defect detection algorithms generally have high defect recognition accuracy. Machine learning algorithms require many relevant datasets for training, so are mainly suitable for defect detection in fixed scenarios [35]. The synthetic aperture focusing algorithm leverages time-delay superposition and fluctuation equation theory to reconstruct defect images predicated on the amplitude or wave velocity of ultrasound. However, it takes a long time when the reconstructed image requires a high resolution [36]. The modal analysis algorithm detects defects by analyzing the modal parameters of concrete structures in a vibration state, utilizing various modal parameters and derived modal information, such as vibration mode, modal strain energy, and structural curvature, as the defect characteristics. This approach facilitates not only the localization of defects but also the visualization of the geometric attributes of the damage. For example, Yasuda et al. used laser ablation of vibrated concrete and LDV to check the tunnel lining health and identify internal concrete defects based on the intrinsic frequency and damping ratio [37,38,39]. Sugimoto et al. [40,41,42] proposed a spatial-spectral entropy-based defect imaging algorithm that uses the spatial-entropy spectrum (SSE) to estimate the defect resonance frequency of the data collected via LDV, and displays the size and location of the defect based on the vibration modes at the defect resonance frequency.

The vibration mode is the vibration shape of the subject under test at a characteristic frequency. In the case of damage detection algorithms based on vibration mode, the detection results are significantly influenced by the precision of the estimated defect characteristic frequencies. At present, the primary challenges in detecting concrete defects via LDV and modal analysis are twofold. (1) Given the rough surface, the light signal reflected and received by the LDV exhibits weak intensity, resulting in the complexity and weakness of the structural vibration response signal characteristics. Concurrently, the measurement and ambient noise interference during the recording process further complicate the identification of defect features. Although the reflective film can enhance the reflectivity of the measured surface [43], it is hardly applicable to all inspection scenarios, such as the inspection of high-rise building facades or high-temperature objects’ surfaces. (2) In the absence of reference mode information for the structure, distinguishing the inherent vibrational frequency of the defect from the numerous characteristic frequencies becomes challenging.

In the case that reference mode information is unavailable, SSE analysis can be used to estimate the resonant frequency of the defect. However, this method is susceptible to the influence of noise. Therefore, it is essential to preprocess the acquired signals prior to SSE analysis. SVD is a typical method in the field of signal processing. It decomposes a signal into a series of singular values and singular vectors, highlighting the characteristics of the original signal. When employing SVD for signal noise reduction, the singular values representing valuable information can be superimposed to recover the valid signal from the noisy one [44]. Since SVD discards some singular values in the denoising process, the denoised signal may be severely distorted, particularly in processing complex vibration signals. In order to make SVD achieve the desired denoising effect, the original signal can be decomposed before SVD denoising, and then SVD denoising may be performed on the decomposed sub-signal, thus alleviating the problem of distortion of the denoised signal. LMD is a non-linear, non-smooth signal analysis method that adaptively decomposes the signal into multiple physically meaningful product function (PF) signals [45]. Compared with wavelet transform [46] and VMD [47], LMD is adaptive and avoids the trouble of parameter setting such as wavelet bases, decomposition layers, and penalty coefficients during decomposition. Compared with EMD [48], LMD can better suppress endpoint effects, and has higher decomposition accuracy. Hence, we combine LMD and SVD as the pre-processing method for the signals acquired via LDV.

This study proposes a concrete internal defect detection algorithm based on LMD-SVD and WSSE. Initially, the algorithm processes the measurement signal of small concrete block with a simulated cavity via LMD-SVD to reduce the LDV-collected signal noise, then employs WSSE to analyze the denoised signals and estimate the internal defects’ intrinsic frequencies. Finally, it calculates the damage factor at each point’s frequency within the scanning plane to characterize the defect size and geometric shape based on the estimated intrinsic frequencies of the defects.

## 2. Theoretical Description and Algorithmic Steps

### 2.1. LMD-SVD Signal Denoising

#### 2.1.1. LMD Principle

The LMD algorithm comprises two iterative loops: the inner and outer loop. The inner loop yields the pure frequency modulation signal, and the outer loop obtains the PF signals. The relationship between the original signal *x*(*t*) and each PF can be expressed using the following equation:

(1) $$x(t)=\sum_{p=1}^{k}{\mathrm{PF}}_{p}(t)+u_{k}(t)$$

where *u_k_*(*t*) denotes the residual signal. In this study, the LMD algorithm steps align with those in reference [49], so the details are not reiterated herein.

Given the resonant frequency range of defects in the collected vibration signals is unknown, we solely address the weakly correlated components and random noise in the signals during denoising, as these components and noise barely represent the vibrational states of the defect. For this reason, we screened and combined the PF signals before executing SVD denoising. The specific steps are as follows:

1. Calculate the Pearson correlation coefficient between the PF signal and the original signal. Discard the PF signal if the correlation coefficient is less than 0.1. This step serves to eliminate the extraneous and irrelevant PF signals produced during the decomposition process.
2. Combining the PF signals with correlation coefficients exceeding 0.7 as PF_C1_ and those between 0.1 and <0.7 as PF_C2_. Subsequently, denoise these two PF components using SVD. This step is to decrease the computational complexity of denoising while preserving the vibration characteristics of the signal.

#### 2.1.2. SVD Principle

Assume that *Y* represents a noisy signal. The denoising procedure of SVD is as follows.

(1) Construct *Y* into a Hankel matrix via delayed embedding. The matrix size is b×c.

(2) $$B_{\mathrm{hankel}}=\left[\begin{array}{llll} y_{1} & y_{2} & \cdots & y_{c}\\ y_{2} & y_{3} & \cdots & y_{c+1}\\ \vdots & \vdots & \vdots & \vdots \\ y_{b} & y_{b+1} & \cdots & y_{N} \end{array}\right]$$

where *N* denotes the length of *Y*, and b=N−c+1. The SVD is calculated according to the following equation.

(3) $$W=USV^{T}$$

where *U* and *V* denote orthogonal matrices, and *V*^T^ denotes the transpose of *V*. *S* denotes the diagonal matrix consisting of singular values, defined as follows:

(4) $$S=\left\{\begin{array}{l} {}[diag(\sigma_{1},\sigma_{2},\ldots ,\sigma_{q}),O]\;b\leq c\\ {\left[diag(\sigma_{1},\sigma_{2},\ldots ,\sigma_{q}),O\right]}^{T}\;\;\;b\;>\;c \end{array}\right.$$

where *O* denotes the zero matrix, q=min(b,c), and $\sigma_{1},\sigma_{2},\ldots ,\sigma_{q}$ denotes the singular values in descending order.

In the theory of singular value decomposition, a larger singular value usually reflects the main components of the signal, and a smaller singular value reflects the weak components of the signal and random noise. Therefore, the principle of SVD denoising involves removing the singular values characterizing noise through a suitable judgment method and reconstructing the denoised signal utilizing the remaining singular values.

#### 2.1.3. Singular Value Selection Algorithm Based on Singular Entropy Increment

To avoid the error in manual selection, we employ an estimation method based on the singular entropy increment to select the order of the singular value reconstruction.

The singular entropy is calculated as follows.

(5) $$H_{qys}(q)=-\sum_{i=1}^{q}E_{i}\log_{2}(E_{i})$$

(6) $$E_{i}=\sigma_{i}^{2}/E$$

(7) $$E=\sum_{i=1}^{q}{\left(\sigma_{i}\right)}^{2}$$

In this paper, the singular entropy increment is defined as follows.

(8) $$D_{\mathrm{diff}}(i{)=\log}_{10}{(H}_{qys}(i+1))-\log_{10}{(H}_{qys}(i))$$

The singular entropy value embodies the complexity of the signal information and expands with the quantity of singular values participating in the signal reconstruction. However, the singular entropy increment stabilizes when the number of contributing singular values achieves a certain threshold, suggesting that the main components of the reconstructed signal have reached saturation. A threshold is established to dictate the order of singular values used for signal reconstruction, calculated as follows:

(9) $$A(i)=\frac{D_{\mathrm{diff}}(i)}{D_{\mathrm{diff}}(i+1)}$$

When *A*(*i*) = 1, the signal reconstructed using the singular values preceding order *i* is deemed the closest to the pure component of the original signal. Set the singular values in the matrix *S* that exceed order *i* to zero to obtain the new diagonal matrix *SD*, and then replace *SD* in Equation (3) to procure the denoised signal. Specifically, *D*_diff_ is rounded to four decimal places in Equation (8).

### 2.2. Theory of WSSE Algorithm

#### 2.2.1. Spectrum Entropy (SE) and Spatial Spectral Entropy (SSE)

**Figure 1:**
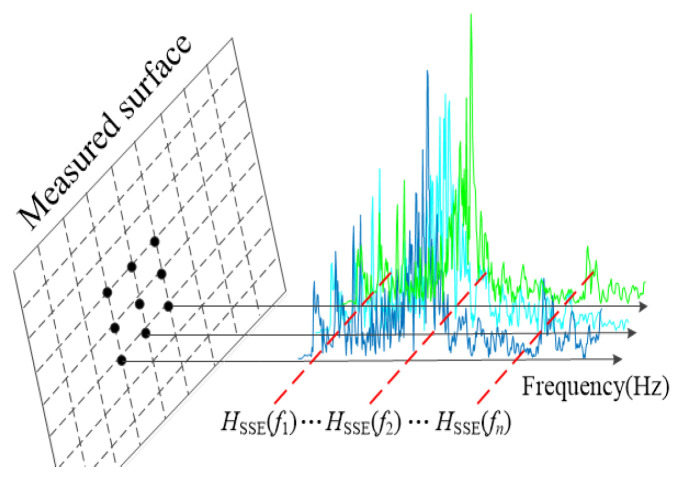
SSE concept diagram.

SE is a feature to used characterize the complexity of a signal [40]. Typically, signals with more concentrated spectral energy (such as vibration and speech) have smaller SE values. On the contrary, signals with spectral characteristics akin to white noise will have larger SE values. The SE is defined as follows [42].

(10) $$H_{\mathrm{SE}}=-\sum_{f}^{}P_{f}\log_{2}P_{f}$$

(11) $$P_{f}=\frac{Q_{f}}{\sum_{f}Q_{f}}$$

where *Q_f_* denotes the amplitude of the signal at frequency *f*. The SSE extends this analysis from independent signals to all measured signals in the two-dimensional scanning plane [42]. Figure 1 shows the conceptual diagram of SSE. As illustrated in Figure 1, following the acquisition of the vibration signal with LDV, the amplitude sequence of the vibration velocity frequency spectrum at the same frequency of all measurement points is considered a probability distribution. To exemplify this, when calculating the SSE of frequency *f*_1_, the frequency spectral amplitude of each measurement point at *f*_1_ is extracted, and then the SE of this amplitude sequence is calculated. The SSE for the remaining frequencies can be obtained with a similar process. The SSE is defined as follows [42].

(12) $$H_{\mathrm{SSE}}(f)=-\frac{\sum_{i=1}^{N_{\mathrm{all}}}P_{i}(f)\log_{2}P_{i}(f)}{\log_{2}(N_{\mathrm{all}})}$$

(13) $$P_{i}(f)=\frac{K_{i}(f)}{\sum_{i=1}^{N_{\mathrm{all}}}K_{i}(f)}$$

where *N*_all_ denotes the number of measurement points in the scanning area. *K_i_*(*f*) denotes the frequency *f* amplitude of the vibration velocity power spectrum at measurement point *i*.

The SSE spectrum characterizes the energy distribution of each frequency component in all measured signals. Since the defect resonant frequency exhibits high vibration peaks only in the defect region, the energy distribution of the defect resonance frequency throughout the entire measurement space demonstrates high non-uniformity. Thus, the SSE value of the defect resonant frequency is smaller than that of other frequencies.

#### 2.2.2. WSSE Algorithm

Although the literature [41] validates the application of the SSE algorithm for estimating the resonant frequencies of defects, the SSE algorithm exhibits poor robustness in relation to noise. When the LDV receives inadequate signal strength, some frequencies may present a high vibration energy under noise influence. In the process of denoising, LMD-SVD may not be able to eliminate these high-energy noise frequencies because they are not considered faint random noise. Since these noise frequency peaks also exhibit a non-uniform characteristic within the measurement space, the SSE algorithm may misjudge these noise frequencies as defective resonant frequencies. Therefore, a WSSE algorithm is proposed to improve the accuracy of defect resonance frequency estimation. In particular, WSSE needs to utilize the correlation information between vibration patterns of the same frequency, thus requiring the acquisition of multiple datasets in the same scanning position. WSSE is defined as follows:

(14) $$H_{\mathrm{WSSE}}(f)=2^{\left|1-W{\left(f\right)}^{2}\right|}\cdot H_{\mathrm{SSE}}(f)$$

where *W*(*f*) denotes the weight function of the SSE at frequency *f*, and is calculated as follows:

(15) $$\left\{\begin{array}{l} W(f)=w_{1}(f)\cdot w_{2}(f)\;\;\;w_{1}(f)<2\cdot w_{2}(f)\\ W(f)=w_{1}(f)-w_{2}(f)\;w_{1}(f)\geq 2\cdot w_{2}(f) \end{array}\right.$$

In Equation (15), *w*_1_ is associated with the degree of correlation of vibration modes of the same frequency at different excitation locations. *w*_2_ is applied to express the vibration level of the vibration pattern at the edge of the scanned area, and its value is related to the energy and the number of edge measurement points. The purpose of using the strategy of conditional judgment to determine the calculation of *W*(*f*) is to reduce the appearance of unreasonable weights of noise frequencies. Since the noise of each measurement is random, the modal vibration pattern of the same frequency noise in each dataset usually has a low correlation. Therefore, when $w_{1}(f)<2\cdot w_{2}(f)$, the subtraction of the two may cause *W*(*f*) to take a relatively large absolute value, which results in a smaller value of the weighted SSE than the original SSE. In this case, using the product form enables the weighting of the noise frequencies to obtain a small value. Conversely, when $w_{1}(f)\geq 2\cdot w_{2}(f)$, the value of WSSE is determined by the difference between *w*_1_ and *w*_2_.

Suppose the scanning grid size is $n_{\mathrm{row}}\times n_{\mathrm{col}}$, the number of measurement points is $N_{\mathrm{grid}}=n_{\mathrm{row}}\times n_{\mathrm{col}}$, and a total of *N*_EP_ datasets are collected. Then, the specific calculation of *w*_1_ is as follows.

(16) $$w_{1}(f)=\bar{\sum_{i}^{N\mathrm{cor}}\rho_{G_{\left(i,1\right)}^{}(n),G_{\left(i,2\right)}^{}(n)}}=\bar{\sum_{i}^{N\mathrm{cor}}\frac{Cov(G_{\left(i,1\right)}^{}(n),G_{\left(i,2\right)}^{}(n))}{\delta_{G_{\left(i,1\right)}^{}(n)}\delta_{G_{\left(i,2\right)}^{}(n)}}}$$

(17) $$N_{\mathrm{cor}}=\frac{N_{\mathrm{EP}}\cdot (N_{\mathrm{EP}}-1)}{2}$$

where *N*_cor_ denotes the number of all combinations of binomial coefficients of *N*_EP_. $G_{\left(i,1\right)}^{}(n)$ and $G_{\left(i,2\right)}^{}(n)$ denote the sequence of vibration amplitudes in the *i*-th binomial coefficient combination at frequency *f*. In other words, *w*_1_ denotes the average of the Pearson correlation coefficients for each set of frequency amplitude sequences.

The specific steps of *w*_2_ are as follows.

1. Compute the average value of the vibration energy at each measurement point at frequency *f* for the *N*_EP_ group data to obtain the average vibration amplitude sequence *G*_mean_. After arranging *G*_mean_ in ascending order, compute the median of the reordered sequence as the threshold *T*_1_.
2. Rearrange *G*_mean_ into a matrix according to the scanned grid size, and define this matrix as *G*_matrix_. Define all the edge points of *G*_matrix_ as a set denoted *ED*_all_, and then calculate the vibration amplitudes of all the measurement points in *ED*_all_ at frequency *f*.
3. Calculate the average vibration amplitudes of all elements in *G*_matrix_ with amplitudes larger than *T*_1_, and record this average value as the threshold *T*_2._ Then, count the number of measurement points with a vibration energy larger than *T*_2_ in *ED*_all_. Denote the selected edge measurement points as a set *of ED*_s_.

Finally, *w*_2_ is calculated according to the following equation.

(18) $$w_{2}(f)=\left\{\begin{array}{ll} 0 & N_{ED}{}_{{}_{s}}<2\\ \frac{D_{s}\cdot N_{ED_{s}}}{D_{\mathrm{all}}\cdot N_{ED_{\mathrm{all}}}\cdot {\left(ED_{\mathrm{Eratio}}\right)}^{2}} & N_{ED_{s}}\geq 2 \end{array}\right.$$

where $N_{ED}{}_{{}_{s}}$ denotes the number of measurement points in *ED*_s_. $N_{ED}{}_{{}_{\mathrm{all}}}$ denotes the number of measurement points in *ED*_all_. *D*_s_ and *D*_all_ denote the maximum pixel distance between two points in *ED*_s_ and *ED*_all_, respectively. The distance of the adjacent measurement points is set to 1.

(19) $$ED_{\mathrm{ratio}}=\frac{E_{s}}{E_{\mathrm{all}}}=\frac{\sum_{j=1}^{N_{ED_{s}}}{\left|g_{1j}\right|}^{2}}{\sum_{j=1}^{N_{ED_{\mathrm{all}}}}{\left|g_{2j}\right|}^{2}}$$

where *ED*_ratio_ denotes the vibration energy ratio of the measured points in *ED*_s_ and *ED*_all_. *g*_1*j*_ and *g*_2*j*_ denote the vibration amplitude of the measured points in *ED*_s_ and *ED*_all_ at frequency *f*, respectively.

### 2.3. LMD-SVD-WSSE Algorithm Flow

The algorithm is divided into five stages. The first stage is the signal collection stage. In this stage, vibration signal datasets from different excitation sites are collected via LDV, and each measurement dataset contains $n_{\mathrm{row}}\times n_{\mathrm{col}}$ signals.

The second stage involves signal denoising. In this stage, the vibration signal is decomposed into a series of PF signals by LMD. The Pearson correlation coefficients are calculated between each PF signal and the original signal. The PF signals are screened and combined according to Section 2.1.1. The processed PF signals are denoised via SVD according to Section 2.1.2 and Section 2.1.3. Finally, the component signals are reconstructed according to Equations (1) and (3) to obtain the denoised dataset.

The third stage represents the defect vibration frequency estimation stage. Herein, the frequency spectrum of each measurement point in the dataset is normalized, and the weighted SSE spectrum is calculated per the method proposed in Section 2.2.2. The defect’s vibration frequency is estimated using the box plot method. The threshold of the box plot is defined as follows.

(20) $$T_{\mathrm{defect}}=Q_{1\mathrm{st}}-(K\cdot IQR)$$

(21) $$IQR=Q_{3\mathrm{st}}-Q_{1\mathrm{st}}$$

where *Q*_1st_ denotes the lower quartile after arranging the elements in the weighted SSE spectrum in ascending order, and *Q*_3st_ denotes the upper quartile of the rearranged sequence. The *K* value is generally set to 1.5–3 in the box plot method. To reduce the possibility of misjudgment of the defect’s inherent frequency, the *K* value is set to 3 in this paper. The threshold *T*_defect_ is utilized to estimate the defect resonant frequency, and the frequencies of the SSE below the threshold *T*_defect_ are considered the defect resonant frequency.

The fourth stage, the construction of the defect map, involves the computation of the defect factor for each measurement point in each dataset, according to the defect resonance frequency estimated in the previous stage. The defect factor is defined as follows.

(22) $$D_{k}=\frac{{\left|e_{k}\right|}^{2}}{\left(mean(\sum_{i=1}^{N_{\mathrm{ep}}}{\left|e_{\mathrm{all}}{}_{i}\right|}^{2})-{\left|e_{k}\right|}^{2}\right)}$$

where *D_k_* denotes the damage factor value at measurement point *k*-th. *e_k_* denotes the amplitude of the *k*-th measurement point at frequency *f*. *e*_all*i*_ denotes the sum of the amplitudes of the frequency spectrum of the data in group *i*. Then, we summed up the defect maps at the same frequency in each dataset, calculating their average maps and obtaining *N*_defect_ acoustic damage images. The *N*_defect_ denotes the number of defect resonance frequencies.

The fifth stage is the defect map fusion stage. Through the previous steps, vibration images of the defect at different frequencies, which describe the defect’s vibration shape at these frequencies, are obtained. These defect maps require fusion to obtain the comprehensive geometric characteristics of the defects. The fusion process is as follows.

Calculate the Pearson correlation coefficient between the *N*_defect_ defect maps. If the correlation coefficients between two defect maps exceed 0.7, they are replaced by their average maps. Otherwise, the two damaged maps are retained. This step is to avoid some vibration shapes being covered up. Ultimately, the processed defect maps are summed to obtain the final damage map.

**Figure 2:**
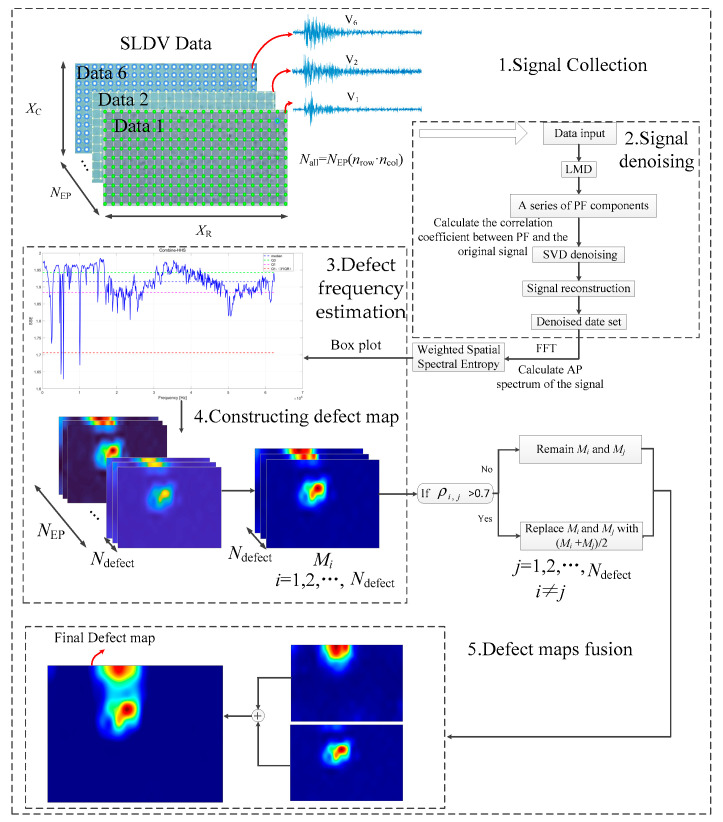
Algorithm flow chart.

Figure 2 shows the flow chart of the LMD-SVD-WSSE algorithm:

## 3. Experimental Environment and Testing

### 3.1. Fabrication of Experimental Test Blocks

**Figure 3:**
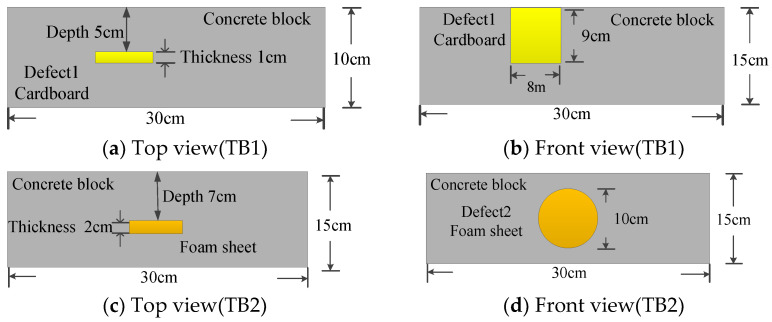
Shape and embedded depth of defect.

The sample blocks in the experiment were made of c30-class concrete. Defects were simulated by embedding cardboard and foam in the test blocks. These blocks were constructed using a customized architectural structure of mixed ordinary Portland cement type I, water, sand, and gravel at 461, 175, 512, and 1252 kg/m^3^. The finished blocks with embedded cardboard (TB1) were 30 cm in length, 10 cm in width and 15 cm in height, and the finished blocks with embedded foam (TB2) were 30 cm in length, 15 cm in width and 15 cm in height. After manufacturing the test blocks, the blocks were maintained for 28 days with natural maintenance. The defect’s spatial location is shown in Figure 3.

### 3.2. Experimental Environment and Test

The experimental system mainly includes an excitation device, a signal acquisition device, and a computer. The excitation device consists of an ultrasonic transmitting controller and an ultrasonic transducer. The ultrasonic transmitting controller emits a ±120 v square wave pulse signal, instigating the ultrasonic transducer (P28F) to produce the ultrasonic signal, with the pulse emission duration established at 10 ms. The signal acquisition device utilized is a PSV-500 LDV produced by Polytec. All algorithms are executed on a computer operating on a 64-bit Windows system, boasting a CPU specification of 2.6 GHz, an Intel Core i7-6700Q with six cores, and a memory specification of 16 GB 2400 MHz DDR4, utilizing Matlab (R2022B).

**Figure 4:**
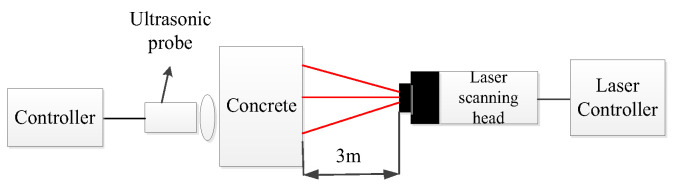
Experimental platform setup.

**Figure 5:**
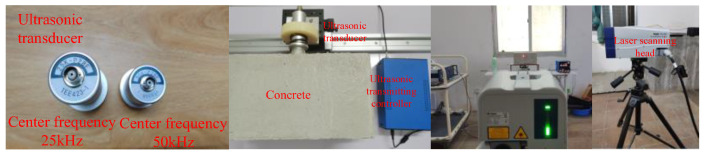
Laboratory equipment.

The experimental platform and equipment are shown in Figure 4 and Figure 5, respectively.

During the experiments, two ultrasonic transducers with differing central frequencies (25 and 50 kHz) were employed to stimulate the test block, in order to evaluate the impact of different excitation source frequencies on the results. The LDV sampling frequency was set at 125 kHz, with the single measurement point sampling duration established at 10 ms, encapsulating 1250 sampling points per signal.

**Figure 6:**
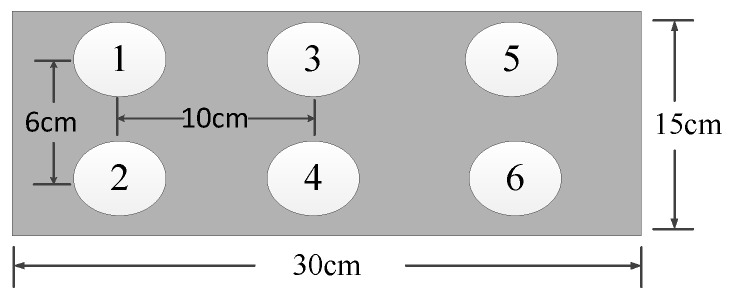
Transducer position (front view).

Due to the ultrasonic transducer’s limited energy and radiation angle, collecting multiple datasets at a fixed excitation location might not accurately identify the defect’s resonant frequency and mode vibration shapes. Hence, excitation at different locations within the scanning area is required to ensure the data from each measurement point effectively reflect the actual internal conditions of the corresponding position within the concrete. Considering the measurement time and effect, six excitation positions were set up within the scanning area, and each excitation position collected one dataset. The position of the transducer excitation for each dataset is shown in Figure 6.

**Figure 7:**
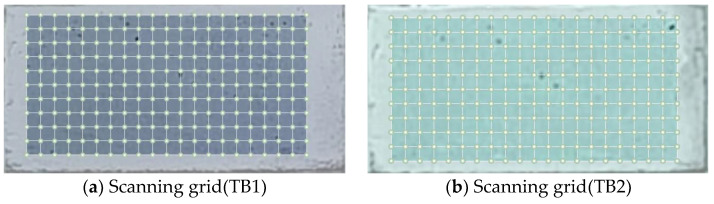
Scan grids of LDV.

The scan grids of TB1 and TB2 are shown in Figure 7. The scanning points for TB1 and TB2 are 231 (11 × 21) and 253 (11 × 23), respectively.

## 4. Experimental Results and Analysis

### 4.1. Measurement Signal Analysis

**Figure 8:**
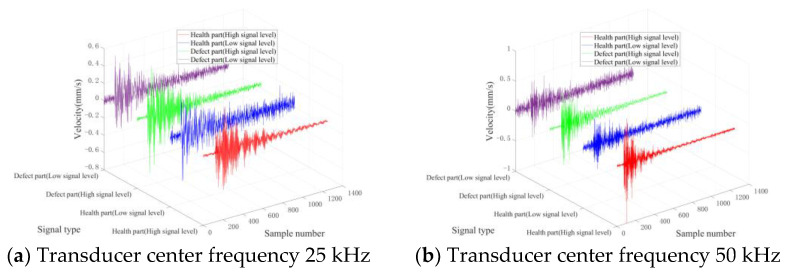
Typical time domain waveforms of TB1.

**Figure 9:**
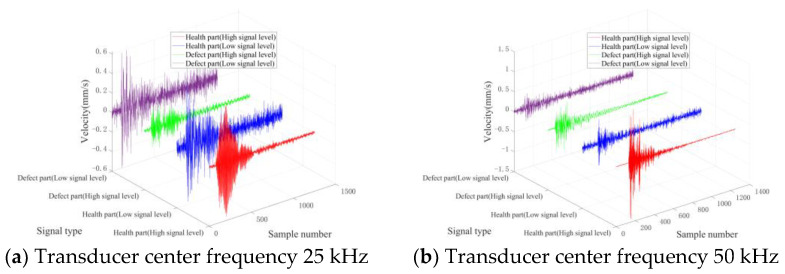
Typical time domain waveforms of TB2.

Figure 8 and Figure 9 present characteristic waveform samples randomly chosen from the two varieties of concrete test blocks. Given that the signal-to-noise ratio (SNR) of the acquired signal cannot be obtained, the SNR is evaluated via the signal level exhibited by the laser scanning head. Signals with level values below a quarter of the signal level indicator’s maximum range are deemed low-level signals.

Figure 8 and Figure 9 reveal that signals emanating from healthy parts are considerably smoother and possess greater amplitudes. Conversely, signals from defective sections display more complex waveforms and reduced amplitudes. Additionally, in the context of low signal levels, both waveform types undergo varying degrees of distortion, thereby complicating the differentiation of the two signal types in the time domain.

**Figure 10:**
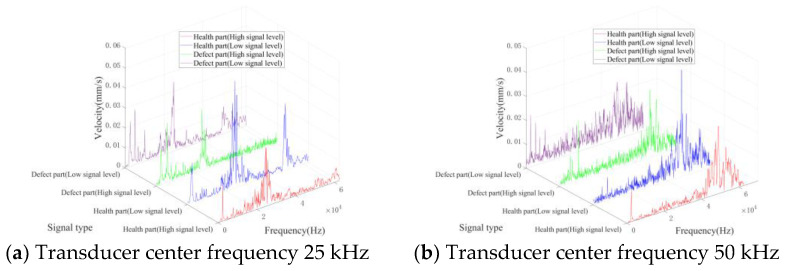
Typical frequency domain waveforms of TB1.

**Figure 11:**
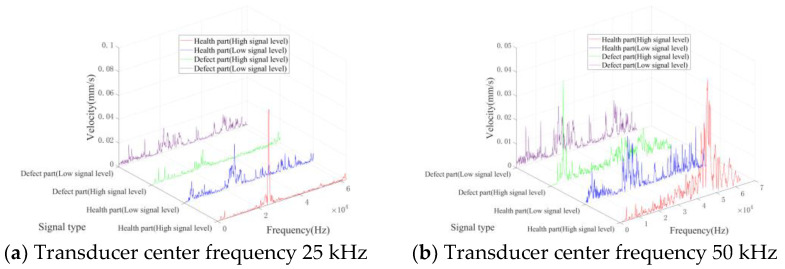
Typical frequency domain waveforms of TB2.

Figure 10 and Figure 11 show the spectrum of the above signal after the Fourier transform. The frequency spectra of all kinds of signals display distinct peaks proximate to the central frequency of the ultrasonic transducer. For signals originating from healthy parts, the spectral energy is primarily concentrated around the vibration peak caused by the ultrasonic transducer, without distinct peaks across various frequency bands. Due to the bending vibration caused by the defect, signals from defective parts exhibit vibration peaks across other frequency bands. In addition, low-level signals possess more complex spectral frequency components compared to high-level signals. Specifically, the signal spectrum of the healthy part also shows multiple vibration peaks under the influence of noise.

**Figure 12:**
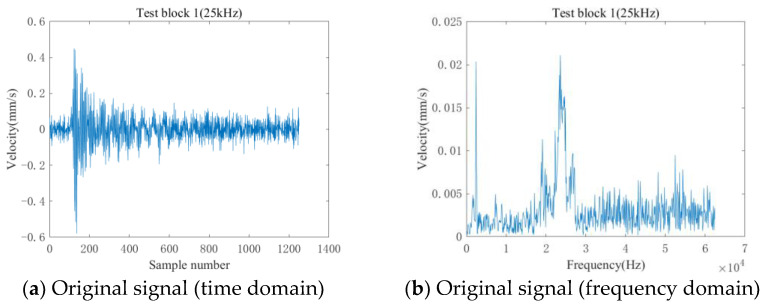

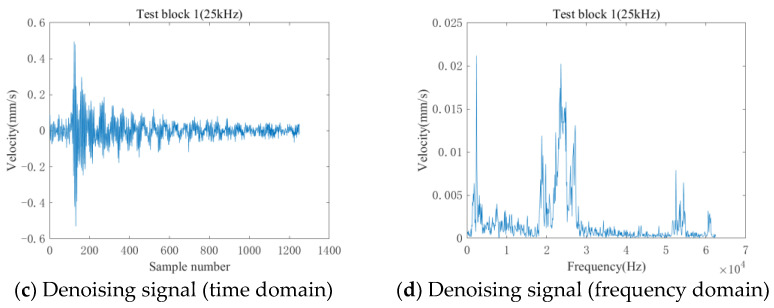
Noise reduction result.

Figure 12 shows the original and denoised waveforms of a vibration signal randomly selected from TB1. As can be seen in Figure 12, the original signal contains many spurious waves, and its spectrum has distinct vibrational peaks in all frequency bands. After denoising via LMD-SVD, the signal’s time-domain waveform is smoother, and the burrs are reduced. In terms of spectrum, the denoised signal retains the principal distinct peaks while effectively suppressing the peaks caused by spurious waves and noise in other frequency bands.

### 4.2. Differences in Compared Defective Resonant Frequency Identification Methods

In this section, the effect of different signal processing algorithms on defect resonant frequency estimation performance was tested on a dataset obtained under 25 kHz transducer excitation. We compared four types of methods, as shown in Table 1. Each method is referred to as Case 1, 2, 3, and 4 in the subsequent text.

Case 1 represents the result of the literature [42] algorithm. Case 2 results from the addition of the LMD-SVD algorithm, based on the literature [42] algorithm. Case 3 represents the outcome of employing WSSE alone. Case 4 exemplifies the result of estimating the defect resonance frequency via the proposed algorithm in Section 2.3.

**Figure 13:**
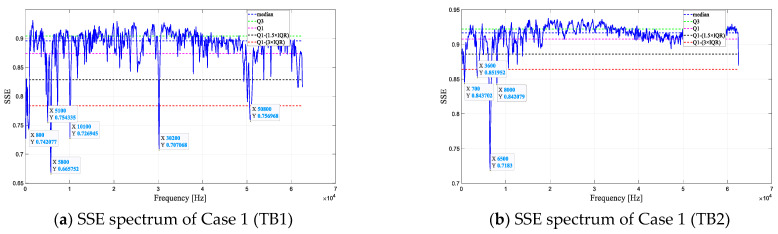

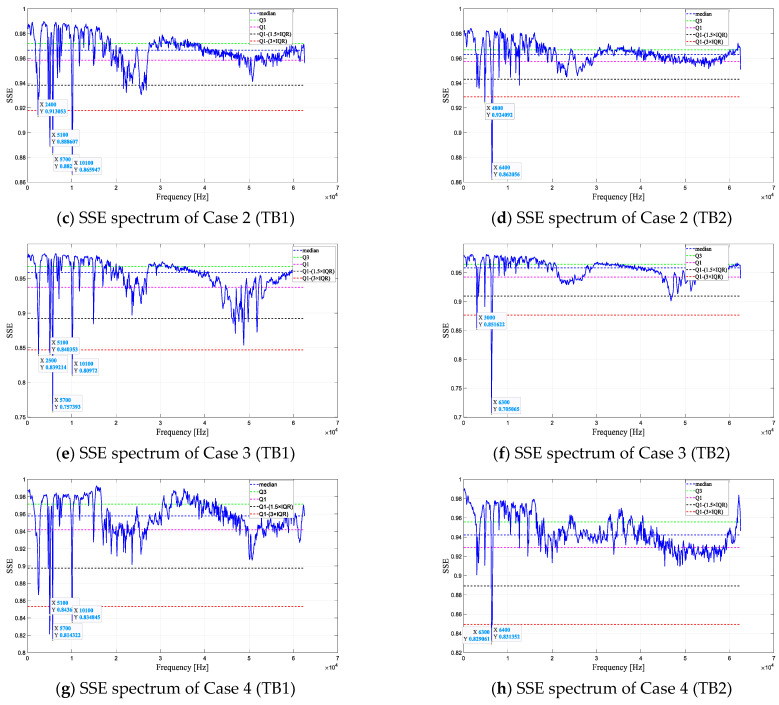
Defect resonant frequency estimation results (25 kHz).

Figure 13 shows the results of defect resonant frequency estimation using four methods. Frequencies below the red dashed line are considered defect resonant frequencies. As depicted in Figure 13, the defect resonant frequencies estimated by the four methods differ. Cases 2 and 3 eliminate some defect resonant frequencies present in Case 1. In the SSE spectra of Case 4, the range of defect resonant frequencies in Cases 2 and 3 is further reduced.

**Figure 14:**
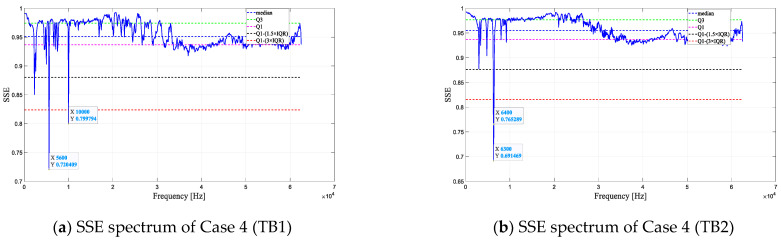
Defect resonant frequency estimation result (50 kHz).

Figure 14 shows the results of defect resonant frequency estimation in Case 4 with a 50 kHz excitation frequency. As shown in Figure 14, the estimated resonant frequencies of the defects are the same using different frequencies of the excitation source. This indicates that changing the excitation source frequency does not affect the actual defect resonant frequency. In addition, 5 kHz was identified as the defect resonant frequency under the excitation source of 25 kHz, yet it was not recognized under the 50 kHz excitation source. This might be due to the 50 kHz excitation source failing to effectively stimulate the vibrational response of the 5 kHz defect.

Table 2 shows the correlation coefficients between the defect maps corresponding to the defect resonant frequencies at a 25 kHz center frequency in TB1.

As can be seen from Table 2, the defect maps at 5100 and 5700 HZ are highly correlated; thus, even though the transducer with a central frequency of 50 kHz inadequately excites the 5 kHz defect vibration frequency, it does not significantly affect the final fused defect map.

**Figure 15:**
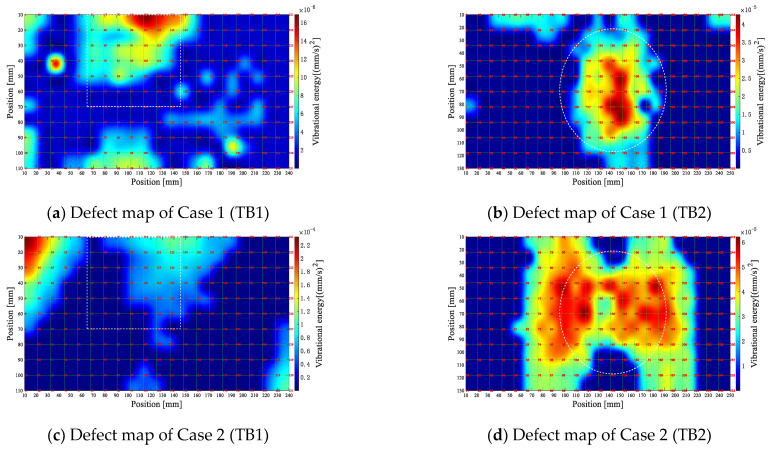

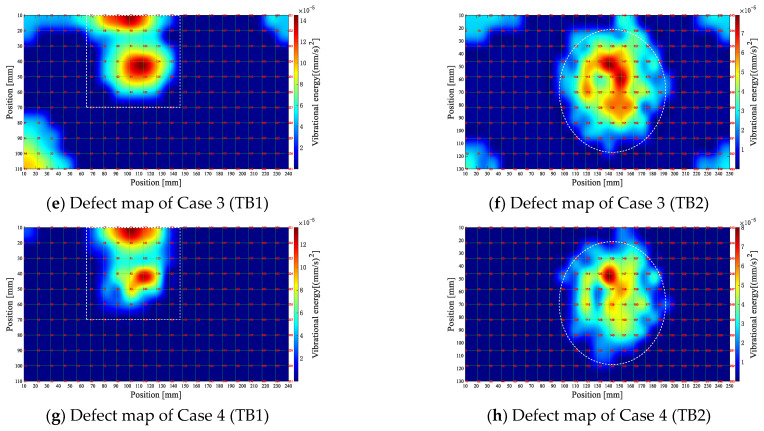
Final defect map of each different case (25 kHz).

Figure 15 displays the final defect maps for Cases 1 to 4 at a 25 kHz center frequency. The white dashed boxes in the figure demarcate the actual size of the defects.

As observed in Figure 15a,b, although the presence of multiple high vibration energies in the defect region for TB1 and TB2 in Case 1 may indicate the possibility of defects, TB1 in Case 1 shows that it is somewhat difficult to accurately identify the location and size of the defect, because relatively high vibration energy points also exist in the non-defective region due to noise interference. From Figure 15c,d, it can be seen that the defect map of Case 2 failed to identify the defects effectively. This indicates that there are still non-defect-induced vibration frequencies among the defect frequencies estimated by Case 2, and the vibration shapes of these frequencies have a significant influence on defect identification. Comparing the defect maps of Case 1 and Case 3 reveals that Case 3’s defect map mitigates noise interference and better accentuates the geometric features of the defects. Nonetheless, some healthy regions in the Case 3 defect map still exhibit elevated vibrational energy, thus impeding internal defect detection. Figure 15g,h illustrate that the defect profile provided by Case 4 most closely resembles the actual defects, eliminating anomalous vibrations in the healthy regions and substantially enhancing the accuracy of defect identification.

**Figure 16:**
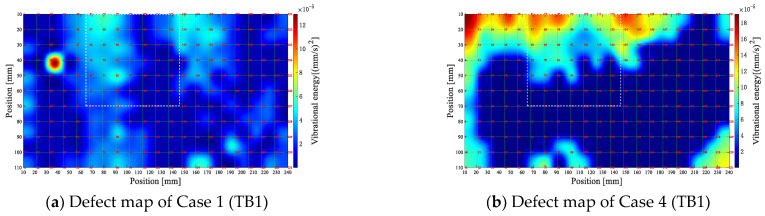
Final defect maps for different cases at the threshold value of *Q*_1st_ − 1.5 *IQR*.

In addition, the defect maps for TB1 at a defect frequency threshold equal to *Q*_1st_ − 1.5 *IQR* are shown in Figure 16 to test the effect of different threshold selections on the recognition performance of the algorithm.

As observed in Figure 16a,b, the noise drowns out the defect map in Case 1, and the vibration level in the defect area is not prominent compared to the healthy region, thus making it difficult to reflect the actual size of the simulated defect. In Case 4, although significant vibration can be observed in the defect region, some areas with higher values are unrelated to the simulated defects. Hence, *K* is more appropriately set to 3 in Equation (20) to minimize the defect’s inherent frequency of misjudgment.

### 4.3. Analysis of the Vibration Shape Characteristics of the Test Block

To ascertain whether the feature frequencies removed by Case 4 are related to defects, we extracted data for TB1 and TB2 at excitation position 3 (center frequency of 25 kHz) and analyzed the modal vibration shapes of the two types of test block using the processing software supplied with the PSV-500 system.

**Figure 17:**
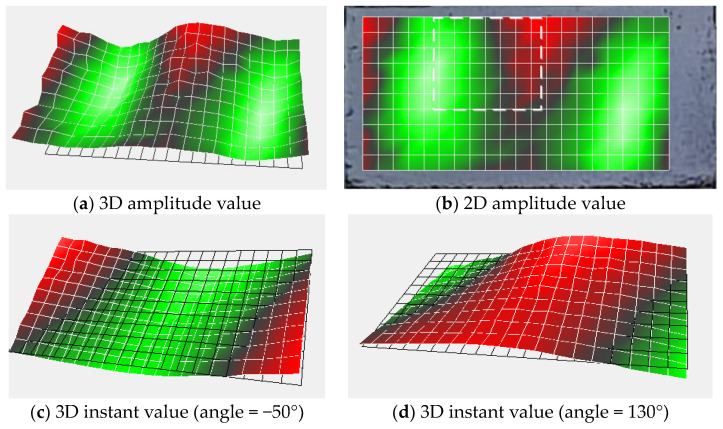
TB1 vibration shape (*f* = 2300 Hz).

**Figure 18:**
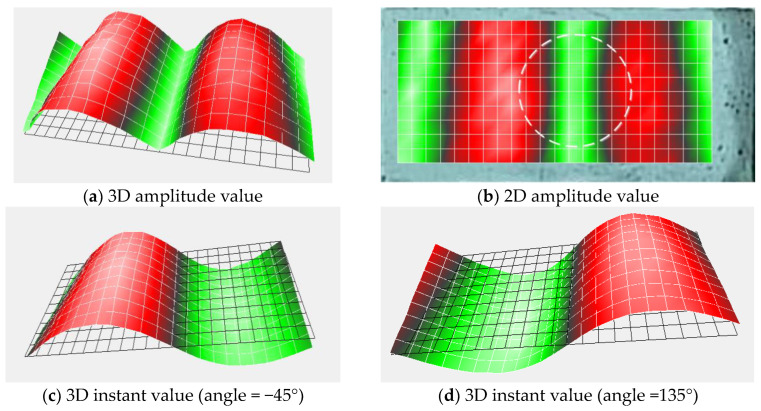
TB2 vibration shape (*f* = 4800 Hz).

Figure 17 and Figure 18 show the TB1 vibration shape at 2300 Hz and the TB2 vibration shape at 4800 Hz, respectively. From these figures, it is apparent that the deformation of the test block at these frequencies encompasses the entire scanning plane, and the vibrational activity of internal defects is unobservable during this deformation. Hence, these vibration modes pertain to the entire structure. At the same time, we observe that the vibration modes of the above frequencies have similar characteristics to the defect vibration modes, implying that these vibration shapes have only a few measurement points with higher energy. Therefore, the original SSE analysis can easily misclassify them as resonant frequencies caused by defects. However, the entire structure’s vibration shape typically exhibits elevated energy at the edge measurement points, whereas the vibration shape of the defect does not necessarily follow this pattern. Only when the defect is located at the edge of the scanning grid does the defect vibration shape have high vibration energy at the edge measurement point. We can use this feature to realize the separation of the defect’s vibration modes and the whole structure’s vibration modes.

Table 3 shows the coefficients *w*_1_, *w*_2_, and the average vibration energy at some characteristic frequencies in Case 4.

As can be seen from Table 3, the *w*_1_ values of 800 Hz and 3600 Hz in TB1 and 700 Hz and 8000 Hz in TB2 are small compared to other frequencies. This illustrates that these frequency vibration shapes have a low correlation at different excitation positions. In Case 2 and Case 3, these frequencies cease to be identified as defect resonant frequencies, suggesting that the vibration shape at these frequencies might originate from noise. Since the defect resonant frequency is an intrinsic structural frequency determined by the structure’s properties, the intrinsic vibration shapes of the structure (including the vibration shapes caused by the entire structure and the vibration shapes caused by the defects) usually have high similarity without changing the position or size of the scanned grid. Therefore, characteristic frequencies with larger *w*_1_ values should be prioritized when performing SSE analysis.

As can be seen from the average vibration energy amplitudes in Table 3, the vibration frequencies (2100 Hz and 2300 Hz in TB1, and 3000 Hz and 4800 Hz in TB2) have high vibration energy, which means LMD-SVD cannot eliminate these frequencies from the spectrum. Once these frequencies are misclassified as defect resonant frequencies to construct the defect map, the accurate defect vibration shapes may be covered. Moreover, it is observable that the vibration frequencies unrelated to defects boast a high *w*_2_ value, whereas the defect resonance frequency exhibits a lower *w*_2_. Therefore, we can use *w*_2_ to estimate whether defects cause the vibration shape of a frequency. We set the weight function *W*(*f*) using *w*_1_ and *w*_2_, and weigh the SSE to separate the resonant frequencies of the defect and the entire structure, thus improving the estimation accuracy of the defect resonant frequencies.

In conclusion, the algorithm proposed in this paper effectively differentiates between frequency components induced by defect resonance and those resulting from noise and overall vibration of concrete block, thereby improving the accuracy of defect resonance frequency estimation. In contrast with the conventional SSE algorithm, it demonstrates an efficacy in reducing erroneous detection in the case of concrete blocks that vibrate as a whole.

## 5. Conclusions

This paper proposes a concrete internal defect detection algorithm, accomplishing concrete internal defect detection with modal analysis and laser vibrometry technology. Experiments were conducted using two concrete blocks containing artificially manufactured cavity defects as the inspection objects. An ultrasonic transducer was utilized to stimulate the specimens at multiple excitation points, with an LDV then employed to capture the vibration velocity signals within a consistent detection region. To augment the precision of LDV defect detection, LMD-SVD was applied to minimize the noise in the acquired signal, followed by the deployment of a WSSE algorithm and a box plot method for the differentiation of eigenfrequencies. The results gathered from these experiments indicate that the proposed algorithm efficaciously distinguishes between the characteristic frequencies of measured defect resonance shapes and other vibration shapes, thereby enhancing the accuracy of estimations of defects in small concrete blocks, compared with preceding SSE methodologies.

In forthcoming research, the focus will be on further evaluating the performance of this algorithm in detecting defects within concrete structures. In addition, optimizing the weight parameter to make it more generalizable is an essential task.

## Figures and Tables

**Table 1:** The classification results of different layers.

Signal Processing Method	LMD+SVD	SSE	WSSE
Case 1	-	√	-
Case 2	√	√	-
Case 3	-	-	√
Case 4	√	-	√

**Table 2:** Correlation coefficients between the defect maps in TB1 (25 kHz).

Frequency (Hz)	Concrete	Correlation Coefficient
5000&5100	TB1	0.9801
5000&5700		0.9116
5000&10,010		0.3745

**Table 3:** Comparison results of *w*_1_, *w*_2_, and mean vibration energy.

Frequency (Hz)	Concrete	*w*_1_	Mean Vibration Energy (mm/s)^2^	*w*_2_
800	TB1	0.0326	2.8 × 10^−9^	0.4792
2100		0.1241	2.6 × 10^−8^	0.6649
2300		0.1767	6.1 × 10^−8^	0.5323
3600		0.0602	5.6 × 10^−9^	0.7576
5100		0.4601	4.8 × 10^−8^	0.1951
5700		0.4096	1.8 × 10^−8^	0.0228
10,010		0.3379	1.6 × 10^−8^	0.0074
700	TB2	0.0628	1.8 × 10^−9^	0.2121
3000		0.2231	1.1 × 10^−8^	0.6266
4800		0.2900	1.3 × 10^−7^	0.1754
6300		0.3805	3.0 × 10^−8^	0
8000		0.0679	9.2 × 10^−9^	0.1220

## Data Availability

The data sets in this study are available on request from the corresponding authors. The data is not publicly available due to privacy concerns.
